# hnRNPA2B1 drives colorectal cancer progression via the circCDYL/EIF4A3/PHF8 axis

**DOI:** 10.1002/kjm2.12943

**Published:** 2025-01-15

**Authors:** Yu‐Kai Sun, Jin‐Fu Wang, Xi‐Wen Sun, Ming Zhang

**Affiliations:** ^1^ Experimental and Clinical Research Center, Charité University Medicine Berlin Berlin Germany; ^2^ Max‐Delbrück‐Center for Molecular Medicine, AG, Translational Oncology of Solid Tumors Berlin Germany; ^3^ Department of General Surgery People's Hospital of Rehabilitation Weifang China; ^4^ Department of Gastrointestinal surgery Linyi People's Hospital Linyi China; ^5^ Department of Gastrointestinal Surgery Center Weifang People's Hospital Weifang China

**Keywords:** circCDYL, colorectal cancer, EIF4A3, hnRNPA2B1, PHF8

## Abstract

The RNA‐binding protein hnRNPA2B1 acts as an m6A reader and plays a role in tumor development. This study investigates the potential mechanism of hnRNPA2B1 in colorectal cancer (CRC) progression. The expression profiles of hnRNPA2B1, circCDYL, and PHF8 in CRC cell lines were analyzed. Following si‐hnRNPA2B1 transfection, CRC cell proliferation, invasion, and migration were evaluated by CCK‐8 and Transwell. CDYL expression was detected after actinomycin D and RNase R treatment. RIP was conducted to assess the enrichment of hnRNPA2B1 and m6A on circCDYL. RIP and RNA pull‐down assays established the interaction between circCDYL and EIF4A3/PHF8. EIF4A3 expression was evaluated using RT‐qPCR and Western blot techniques. hnRNPA2B1 and PHF8 displayed high expression levels, whereas circCDYL showed low expression levels in colorectal cancer cells. Inhibition of hnRNPA2B1 reduced CRC cell proliferation, migration, and invasion. hnRNPA2B1 mechanistically elevated the m6A level of circCDYL while decreasing its expression, which in turn reduced the binding of circCDYL to EIF4A3 and enhanced PHF8 expression. In summary, hnRNPA2B1‐mediated m6A modification decreases circCDYL expression, which inhibits the interaction of circCDYL with EIF4A3, enhances PHF8 expression, and ultimately facilitates CRC progression.

## 1 INTRODUCTION

Colorectal cancer (CRC) is a frequently diagnosed malignancy worldwide and, in particular, the rising incidence of CRC in young individuals deserves clinical attention.^1^ Initiation, promotion, progression, and metastasis are the four steps that naturally lead to the development of CRC.^2^ The main cause of CRC mortality is metastasis, further accelerated by the cross‐talk between abnormally active signaling pathways.^3^ Although the treatment options for both local and advanced CRC have improved due to advances in pathophysiology, the overall survival rate for patients with metastasized disease remains poor.^4^ Therefore, more clarification of the mechanism of CRC progression is required to enable rapid detection and efficient therapy.

As a heterogeneous nuclear ribonucleoprotein (hnRNP) family member, hnRNPA2B1 is implicated in multiple aspects of RNA biology, such as alternative splicing, mRNA processing, and transcriptional and translational regulation.^5^ hnRNPA2B1 is acknowledged as a critical oncogenic driver in various cancers including but not limited to multiple myeloma,^6^ breast cancer,^7^ and non‐small‐cell lung cancer.^8^ hnRNPA2B1 is abundantly expressed in colon cancer and significantly expedites colon cancer growth in vitro and in vivo.^9^ Knockdown of hnRNPA2B1 abates the enhanced CRC cell migration and invasion caused by H19.^10^ Although it has been revealed that hnRNPA2B1 is overexpressed in several malignancies, the precise regulation mechanism of hnRNPA2B1 in CRCR remains unclear.

Circular RNAs (circRNAs) are a group of recently identified non‐coding RNAs formed by covalently closed loops via back splicing.^11^ circCDYL dysregulation has been observed in a wide range of malignancies, including breast cancer,^12^ bladder cancer,^13^ and colon cancer.^14^ CircCDYL overexpression can repress colon cancer cell growth and migration by diminishing miR‐150‐5p expression.^14^ N6‐methyladenosine (m6A) is one of the most common internal modifications observed in coding and non‐coding RNAs, including circRNAs.^15^ Aberrant m6A profiles have been associated with carcinogenesis and progression of CRC.^16^ While substantial evidence has revealed the relationship between m6A modification, circRNAs, and CRC, the impact of m6A modification on the initiation and progression of CRC through the modification of circCDYL remains unclear.

The RNA‐binding protein hnRNPA2B1 has been shown to bind m6A‐modified RNAs, acting as a m6A reader.^17^ hnRNPA2B1 is elevated in CRC tissues, and its elevation is significantly associated with the clinical pathological features and survival prognosis of CRC.^18^ The increase of miR‐92a, derived from hnRNPA2B1‐m6A alteration, is a potential noninvasive diagnostic biomarker for CRC.^19^ hnRNPA2B1 facilitates the incorporation of miR‐934 into exosomes of CRC cells, hence triggering M2 polarization in macrophages to enhance liver metastasis of CRC.^20^ hnRNPA2B1 interacts with MIR100HG to facilitate epithelial‐to‐mesenchymal transition (EMT) in CRC via m6A‐dependent stabilization of TCF7L2 mRNA.^21^ The role of hnRNPA2B1‐mediated m6A modification in regulating circCDYL in CRC remains unclear. Thus, examining the regulatory mechanism of hnRNPA2B1‐mediated m6A modification in the context of CRC development is essential. This study investigates how hnRNPA2B1 promotes CRC growth and metastasis through the circCDYL/EIF4A3/PHF8 axis, identifying potential therapeutic targets for CRC treatment.

## 2 MATERIALS AND METHODS

### 2.1 Cell culture and treatment

Human CC cell lines (SW620, SW480, HCT‐8, and HCT‐116) and human colonic mucosal epithelial cell line (NCM460) were purchased from American Type Culture Collection (ATCC, Manassas, VA, USA) and cultured in RPMI1640 medium (Gibco, Grand Island, NY, USA) containing 10% fetal bovine serum (FBS) (Gibco) and 1% penicillin/streptomycin (Gibco) at 37°C with 5% CO_2_.

Small interfering RNA (siRNA) oligonucleotides targeting hnRNPA2B1 or circCDYL or eukaryotic translation initiation factor 4A3 (EIF4A3) and negative control were designed and synthesized by GenePharm (Shanghai, China). Full‐length plant homeodomain finger protein 8 (PHF8) was subcloned into pcDNA3.1, using empty pcDNA3.1 as a control. Upon reaching an 80% confluence rate, the constructed siRNAs or plasmids were transfected into HCT‐116 and SW480 cells employing Lipofectamine 2000 reagent (Invitrogen, Carlsbad, CA, USA). The efficiency of gene intervention was detected by RT‐qPCR or Western blot after 48 h.

### 2.2 Cell counting kit‐8 (CCK‐8) assay

The cell proliferation was evaluated using a CCK‐8 assay kit (Sigma‐Aldrich, Merck KGaA, Darmstadt, Germany). Cells were seeded in 96‐well plates at a density of 3 × 10^3^ cells per well. At 24, 48, and 72 h, 10 μL of CCK‐8 solution was added to each well and incubated in the dark for 2 h. Absorbance at 450 nm was recorded every 24 h using a microplate reader (BioTek Instruments, Winooski, VT, USA).

### 2.3 EdU staining

After 24 h of culture in 96‐well plates (1.5 × 10^5^), the cells were fixed with 4% paraformaldehyde at room temperature for 30 min, treated with 50 mM EdU solution for 2 h, and then sealed with Apollo dye solution and Hoechst 33342. A fluorescent microscope (Nikon, Tokyo, Japan) was used to take pictures, and randomly selected fields of view were used to determine the proportion of EdU‐positive cells.

### 2.4 Transwell

A Transwell assay assessed the migration and invasion of cells (5 × 10^4^). The migration and invasion experiments were conducted with the apical chamber coated/uncoated Matrigel (BD Biosciences, Franklin Lakes, NJ, USA), and 10% FBS was added into the basolateral chamber. Following a 24 h incubation at 37°C, the cells were carefully removed using cotton swabs. The migrating or invaded cells were preserved with methanol and stained using crystal violet. The migrated or invaded cells were finally examined using a microscope (Olympus Corp, Tokyo, Japan).

### 2.5 RNase R treatment

The stability of circCDYL was evaluated by extracting the total RNA of cells using the TRIzol reagent (Invitrogen, Carlsbad, CA, USA) and then incubating 3 μg of total RNA with 10 U RNase R (20 U/μL; Epicenter, Madison, WI, USA) at 37°C for 30 min. The RNase R was then deactivated by treating the cells at 75°C for 10 min.

### 2.6 Actinomycin D treatment

The cells were seeded into 6‐well plates. After 4 h, the cells were treated with actinomycin D (5 μg/mL; Sigma‐Aldrich) and collected after 0, 1, 4, 8, and 12 h of treatment.

### 2.7 RNA immunoprecipitation (RIP)

A Magna RIP RNA binding protein immunoprecipitation kit (Millipore, Bedford, MA, USA) was used to perform the RIP experiment on the cells. Abcam (Cambridge, MA, USA) supplied the IgG antibody (ab172730) and the anti‐hnRNPA2B1 antibody (ab183654). To detect RIP containing m6A, an anti‐m6A antibody (ab208577, Abcam) was used.

### 2.8 RNA pull‐down

The cells were cross‐linked using 0.3% formaldehyde and quenched with a glycine solution (Millipore). The biotin‐labeled circCDYL probe and the control probe were produced by Sangon Biotech in Shanghai, China. Biotin‐labeled RNA pull‐down was conducted following the guidelines of the EZ‐Magna ChIRP RNA Interaction Kit (Millipore).

### 2.9 Real‐time reverse transcriptase‐polymerase chain reaction (RT‐qPCR)

The total RNA was extracted using TRIzol reagent (Invitrogen). The quantity and quality of extracted RNA were assessed using the NanoDrop 2000c (Thermo Fisher Scientific Inc., Waltham, MA, USA). RNA samples displaying A260/A280 ratios >2 were selected for quantitative analysis. The Revert Aid First Strand cDNA Synthesis Kit (ThermoFisher) was used for cDNA synthesis. Real‐time quantitative PCR was conducted employing SYBR® Premix Ex TaqTM II (Takara Bio Inc., Shiga, Japan). The candidate gene expression was evaluated on the ABI 7900HT system (Applied Biosystems, Inc., Carlsbad, CA, USA). PCR primers are shown in Table 1. The relative expression of the gene was calculated by the 2^−ΔΔCt^ method,^22^ with glyceraldehyde‐3‐phosphate dehydrogenase (GAPDH) as the internal reference.

**TABLE 1:** PCR primer sequences.

Name	Sequence (5′–3′)
hnRNPA2B1	F: GGAGTGGAAGAGGAGGCAAC
	R: ATCCCCAAATCCACGTCCAC
circCDYL	F: CTTAGCTGTTAACGGGAAA
	R: CTGTTGAAGTCGTGGATGT
CDYL	F: GACGACAGAAGAGACCAGCC
	R: AAGCCATCCTGCTTCCTGAC
EIF4A3	F: CAGCAACGAGCAATCAAGCA
	R: GAGCAAGCAGCCCCTGAATA
PHF8	F: TCCAAATCTCGGCGAACCAA
	R: GTCGCCTTCTCCTTTCCCAA
GAPDH	F: GATGCTGGCGCTGAGTACG
	R: GCTAAGCAGTTGGTGGTGC

Abbreviations: CDYL, chromodomain Y‐like; EIF4A3, eukaryotic translation initiation factor 4A3; GAPDH, glyceraldehyde‐3‐phosphate dehydrogenase; hnRNPA2B1, heterogeneous nuclear ribonucleoprotein A2B1; PHF8, plant homeodomain finger protein 8.

### 2.10 Western blot

The cells were collected, and the protein was extracted in cell lysis buffer (Beyotime, Shanghai, China). After centrifugation (14,000 g, 5 min, 4°C), the obtained protein (15–30 μg) was loaded to SDS‐PAGE and transferred onto nitrocellulose membranes (Millipore). Then, the membranes were blocked with 5% skimmed milk and incubated with the primary antibodies hnRNPA2B1 (ab259894, 1:1000, Abcam), EIF4A3 (ab180573, 1:1000, Abcam), PHF8 (ab280887, 1:1000, Abcam), and β‐actin (ab8227, 1:1000, Abcam) at 4°C overnight, followed by incubation with the secondary antibody (ab205718, 1:2000, Abcam) at 37°C for 2 h. The target proteins were visualized using an enhanced chemiluminescence reagent (Millipore).

### 2.11 Statistical analysis

Data analysis and map plotting were performed using SPSS 21.0 (IBM Corp., Armonk, NY, USA) and GraphPad Prism 8.0 (GraphPad Software Inc., San Diego, CA, USA). The data were analyzed for normal distribution and variance homogeneity. The *t*‐test was used for comparisons between the two groups. At the same time, one‐way or two‐way analysis of variance (ANOVA) was applied for comparisons among multiple groups, adhering to Tukey's multiple comparison test. A *p* value of <0.05 signifies a statistically significant difference.

## 3 RESULTS

### 3.1 hnRNPA2B1 promotes proliferation/migration/invasion of CRC cells

**FIGURE 1:**
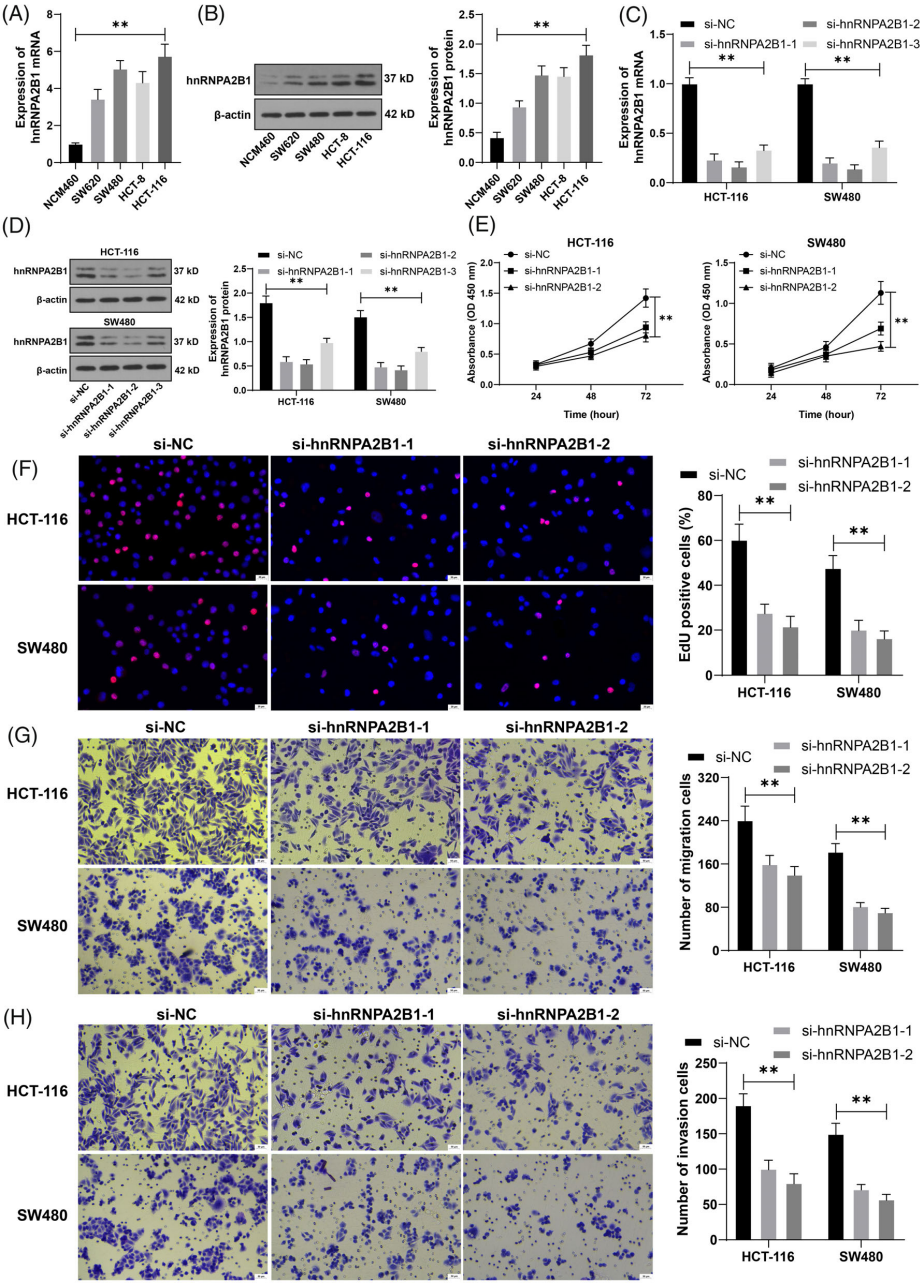
hnRNPA2B1 promotes proliferation/migration/invasion of CRC cells. (A,B) hnRNPA2B1 expression in each cell line was detected by RT‐qPCR and Western blot. Three si‐RNAs targeting hnRNPA2B1 were transfected into HCT‐116 and SW480 cells, respectively, with NC‐siRNA (si‐NC) as a negative control. (C,D) hnRNPA2B1 expression in each group of cells was detected by RT‐qPCR and western blot. (E) Cell proliferation was evaluated using a CCK‐8 assay. (F) The percentage of EdU‐positive cells was detected by EdU staining. (G,H) Transwell detected cell invasion and migration. The cell experiments were repeated three times independently. Data are presented as mean ± standard deviation. Data in panels (A,B) were analyzed by one‐way ANOVA and data in panels (C–H) were analyzed by two‐way ANOVA, followed by Tukey's multiple comparisons tests. **p < 0.01.

hnRNPA2B1 is highly expressed in colorectal cancer.^9^, ^23^ The results revealed that hnRNPA2B1 expression in CRC cell lines was significantly higher than in NCM460 cells (*p* < 0.05, Figure 1A,B). This study subsequently selected HCT‐116 and SW480 cells, which show elevated expression levels of hnRNPA2B1, for further experimentation. The si‐hnRNPA2B1 was transfected into the two cell strains, successfully reducing the expression of hnRNPA2B1 (*p* < 0.05, Figure 1C,D) and selecting two siRNAs with better transfection efficiency for verification. Compared with si‐NC treatment, si‐hnRNPA2B1 treatment abated cell proliferation activity and declined the percentage of EdU‐positive cells (*p* < 0.05, Figure 1E,F). After a silence of hnRNPA2B1, the migration and invasion of cells were weakened (*p* < 0.05, Figure 1G,H). The results indicated that hnRNPA2B1 was significantly expressed in CRC cells, and the silencing of hnRNPA2B1 inhibited the proliferation, migration, and invasion of CRC cells.

### 3.2 hnRNPA2B1 elevates the circCDYL m6A level and reduces circCDYL expression

**FIGURE 2:**
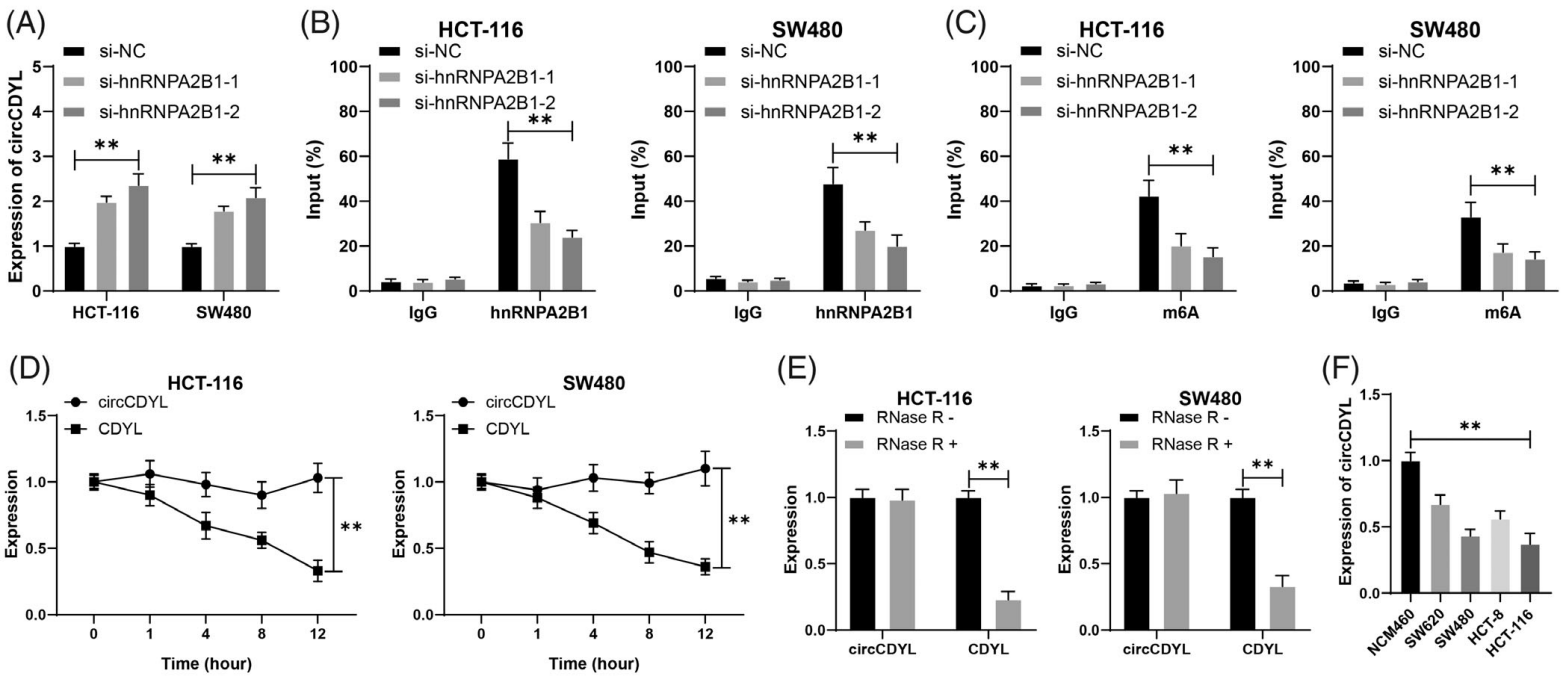
hnRNPA2B1 elevates circCDYL m6A level and reduces circCDYL expression. (A) circCDYL expression in cells was detected by RT‐qPCR. (B,C) The enrichment of hnRNPA2B1 and m6A on circCDYL was analyzed by RIP. (D,E) After actinomycin D and RNase R treatment, circCDYL and CDYL expressions in cells were detected by RT‐qPCR. (F) CircCDYL expression in each cell line was detected by RT‐qPCR. The cell experiments were repeated three times independently. Data are presented as mean ± standard deviation. Data in panels (A,F) were analyzed by one‐way ANOVA and data in panels (B–E) were analyzed by two‐way ANOVA, followed by Tukey's multiple comparisons tests. **p < 0.01.

Inhibition reduces the m6A level of MEG3 but increases its mRNA level.^8^ m6A modification exists in circCDYL, and circCDYL expression is reduced in CRC.^14^, ^24^ CircCDYL is hypothesized to function as a downstream pathway of hnRNPA2B1. This study initially assessed the impact of hnRNPA2B1 on the expression of circCDYL and observed that the silencing of hnRNPA2B1 elevated the expression of circCDYL (*p* < 0.05, Figure 2A). An RIP assay was used with hnRNPA2B1 antibody in CRC cells to investigate the binding connection between hnRNPA2B1 and circCDYL. As shown in Figure 2B, hnRNPA2B1 bound more circCDYL than IgG (*p* < 0.05), while compared with the si‐NC group, the si‐hnRNPA2B1 group pulled down less circCDYL (*p* < 0.05, Figure 2B). Subsequently, an investigation was conducted to determine if the regulation of circCDYL by hnRNPA2B1 was dependent upon m6A alteration, revealing that the m6A level of circCDYL in cells was significantly diminished following si‐hnRNPA2B1 treatment (*p* < 0.05, Figure 2C). Further, circCDYL showed certain stability (*p* < 0.05, Figure 2D,E), and its expression was decreased in CRC cell lines (*p* < 0.05, Figure 2F). In summary, hnRNPA2B1 increased the m6A level of circCDYL while reducing the expression of circCDYL.

### 3.3 Inhibition of circCDYL reduces the inhibitory effect of hnRNPA2B1 silencing on CRC cells

**FIGURE 3:**
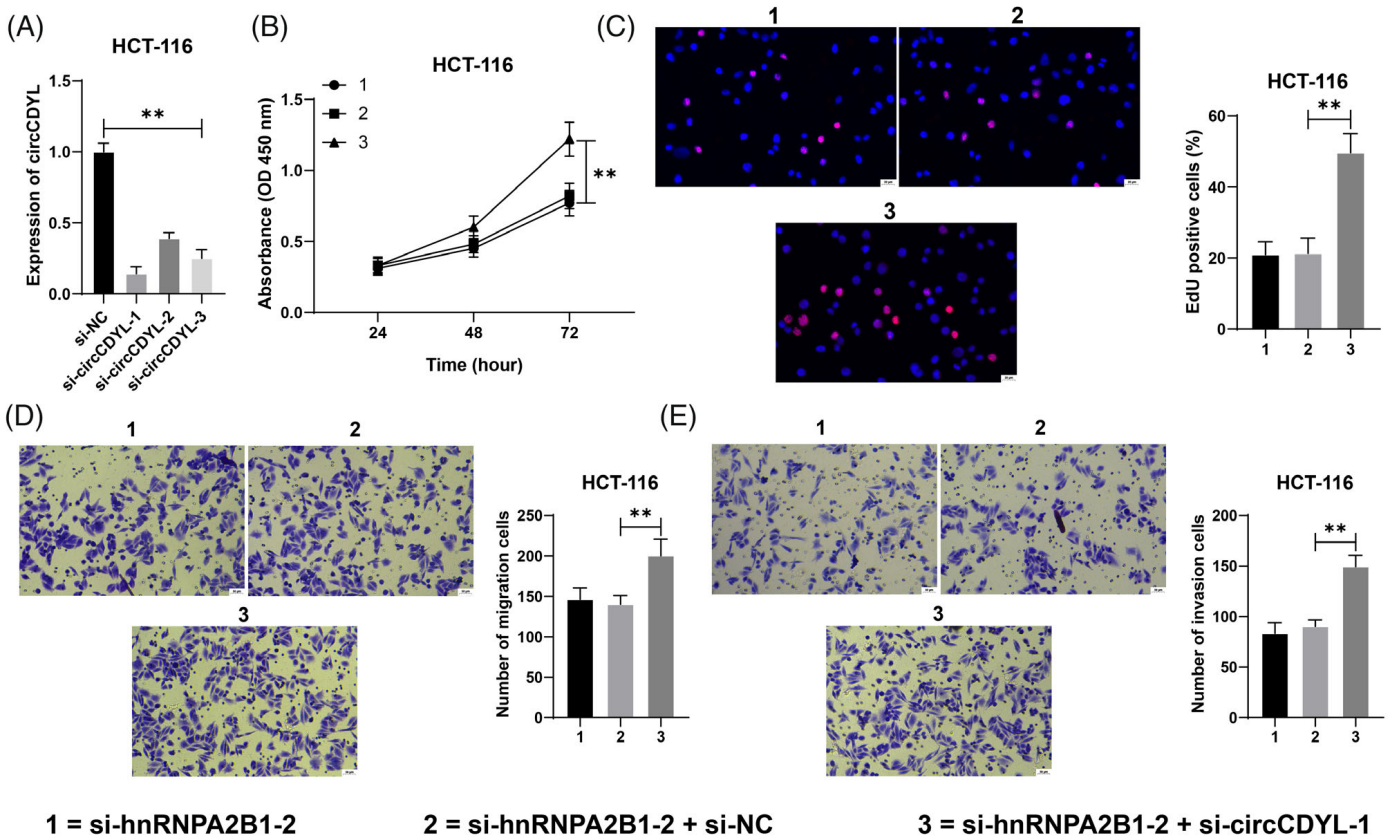
Inhibition of circCDYL reduces the inhibitory effect of hnRNPA2B1 silencing on CRC cells. Three si‐RNAs targeting circCDYL were transfected into HCT‐116 cells, respectively, with NC siRNA (si‐NC) as a negative control. (A) circCDYL expression in cells was detected by RT‐qPCR. (B) Cell proliferation was evaluated using a CCK‐8 assay. (C) The percentage of EdU‐positive cells was detected by EdU staining. (D,E) Transwell detected cell invasion and migration. The cell experiments were repeated three times independently. Data are presented as mean ± standard deviation. Data in panels (A,C–E) were analyzed by one‐way ANOVA and data in panel (B) by two‐way ANOVA, followed by Tukey's multiple comparisons tests. **p < 0.01.

This study effectively decreased circCDYL expression in HCT‐116 cells by transfecting si‐circCDYL, then selecting si‐circCDYL‐1, which showed higher transfection efficiency, for a combination experiment with si‐hnRNPA2B1‐2 (*p* < 0.05, Figure 3A). Silence of circCDYL led to enhanced proliferation activity, increased percentage of EdU‐positive cells (*p* < 0.05, Figure 3B,C), and augmented cell migration and invasion (*p* < 0.05, Figure 3D,E). According to these findings, circCDYL inhibition decreased the inhibitory effect of hnRNPA2B1 silencing on CRC cells.

### 3.4 circCDYL binds to EIF4A3 to inhibit PHF8 expression

**FIGURE 4:**
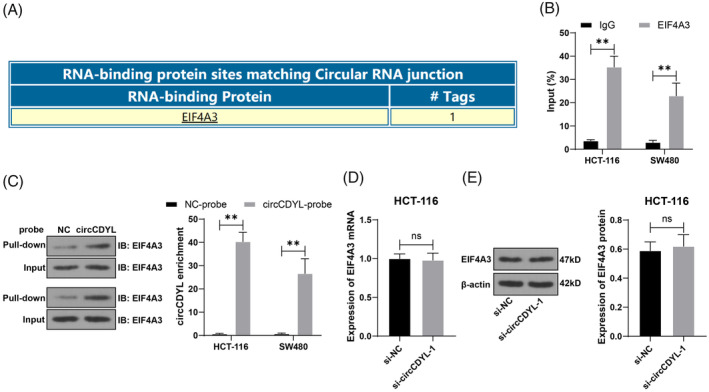
circCDYL binds to EIF4A3. (A) The binding between circCDYL and EIF4A3 was predicted through the Circintractome database. (B,C) RIP and RNA pull‐down verified the binding relationship between circCDYL and EIF4A3. (D,E) The effect of circCDYL on EIF4A3 expression was detected by RT‐qPCR and Western blot. The cell experiments were repeated three times independently. Data are presented as mean ± standard deviation. Data in panels (D,E) were analyzed using a *t*‐test, and data in panels (B,C) were analyzed using two‐way ANOVA, followed by Tukey's multiple comparisons tests. **p < 0.01.

circRNA can bind to the RNA‐binding protein HuR to reduce the interaction between HuR and mRNA, thereby inhibiting mRNA expression.^25^ Through the Circintractome database (https://circinteractome.nia.nih.gov/rna_binding_protein.html) prediction,^26^ it was found that circCDYL could bind to the RNA‐binding protein EIF4A3 (Figure 4A). The interaction between circCDYL and EIF4A3 was verified using the RIP assay. CircCDYL was more abundant in EIF4A3 immunoprecipitation than IgG (*p* < 0.05, Figure 4B). RNA pull‐down data also showed that EIF4A3 was significantly enriched in the circCDYL probe group (*p* < 0.05, Figure 4C). Changes in circCDYL expression did not affect the expression of EIF4A3 (*p* > 0.05, Figure 4D,E). In brief, our data suggested that circCDYL could bind to EIF4A3.

**FIGURE 5:**
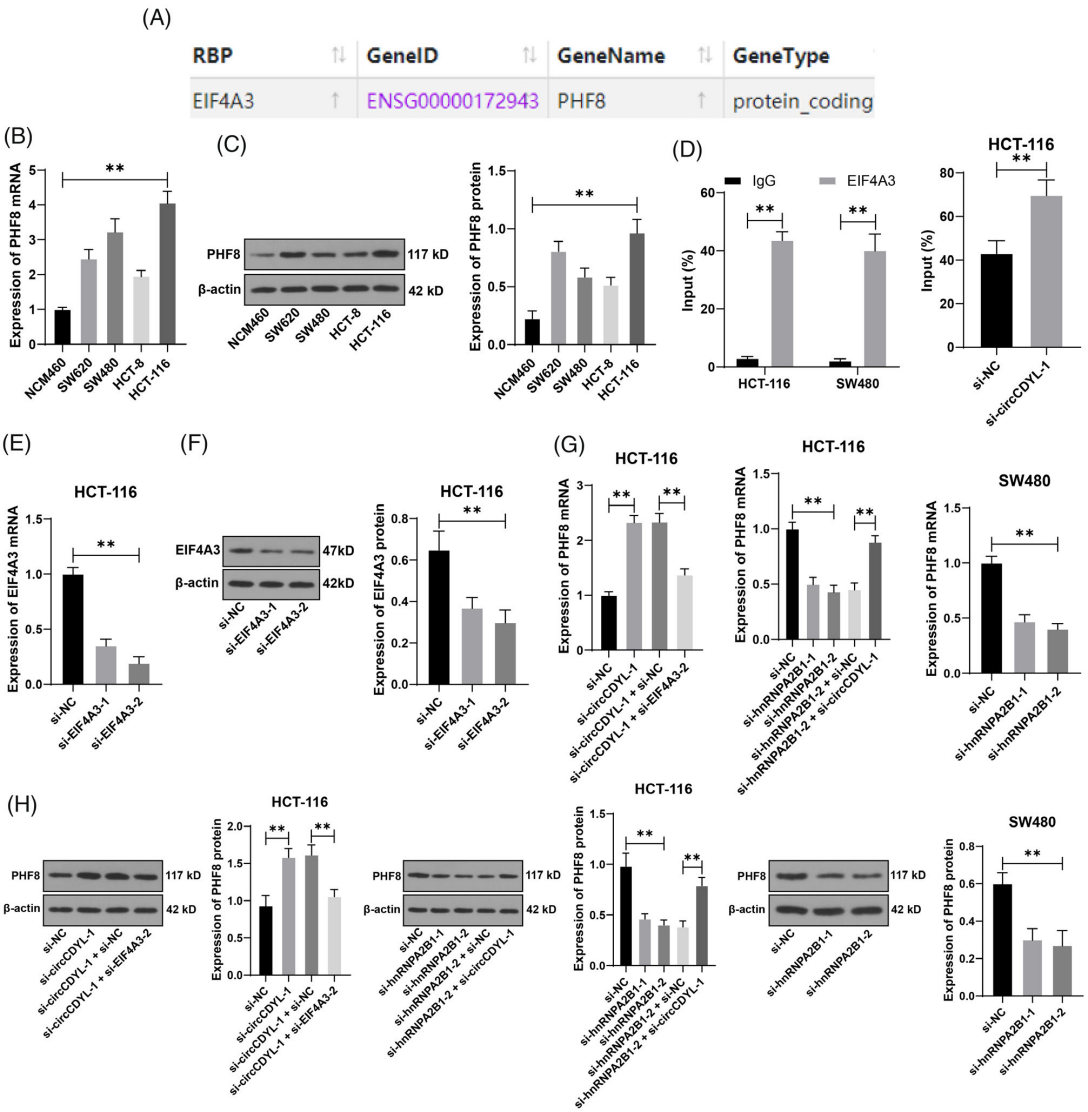
circCDYL binds to EIF4A3 to inhibit PHF8 expression. (A) The binding between EIF4A3 and PHF8 was predicted through the Starbase database. RT‐qPCR and Western blot detected (B,C) PHF8 expression in each cell line. (D) The binding relationship between EIF4A3 and PHF8 was verified by RIP. Two si‐RNAs targeting EIF4A3 were transfected into HCT‐116 cells, respectively, with NC siRNA (si‐NC) as a negative control. (E,F) EIF4A3 expression in cells was detected by RT‐qPCR and Western blot. (G,H) PHF8 expression in cells was detected by RT‐qPCR and Western blot. The cell experiments were repeated three times independently. Data are presented as mean ± standard deviation. Data in penal D (right) were analyzed using the *t*‐test. Data in panels (B–C,E–H) were analyzed by one‐way ANOVA and data in panel (D) (left) were analyzed by two‐way ANOVA, followed by Tukey's multiple comparisons tests. **p < 0.01.

Starbase database (http://starbase.sysu.edu.cn/index.php)^27^ prediction showed that EIF4A3 could bind to PHF8 (Figure 5A). PHF8 expression is elevated in CRC,^28^, ^29^ consistent with our results (*p* < 0.05, Figure 5B,C). This study employed the EIF4A3 antibody for RIP analysis and discovered that PHF8 mRNA could co‐precipitate with EIF4A3 (*p* < 0.05, Figure 5D). The enrichment of PHF8 mRNA in EIF4A3 precipitation was increased due to the knockdown of circCDYL (*p* < 0.05, Figure 5D). To ascertain that circCDYL influenced PHF8 expression through its interaction with EIF4A3, the expression of EIF4A3 in cells was diminished, and si‐EIF4A3‐2, exhibiting improved intervention efficacy, was selected for subsequent investigations (*p* < 0.01, Figure 5E,F). It was found that the expression of PHF8 was increased after a silence of circCDYL but decreased after a silence of EIF4A3 (*p* < 0.01, Figure 5G,H). Moreover, the expression of PHF8 decreased after a silence of hnRNPA2B1 but increased after a silence of circCDYL (*p* < 0.01, Figure 5G,H). The results collectively suggest that the interaction between circCDYL and EIF4A3 suppresses the expression of PHF8.

### 3.5 Overexpression of PHF8 reduces the inhibitory effect of hnRNPA2B1 silencing on CRC cells

**FIGURE 6:**
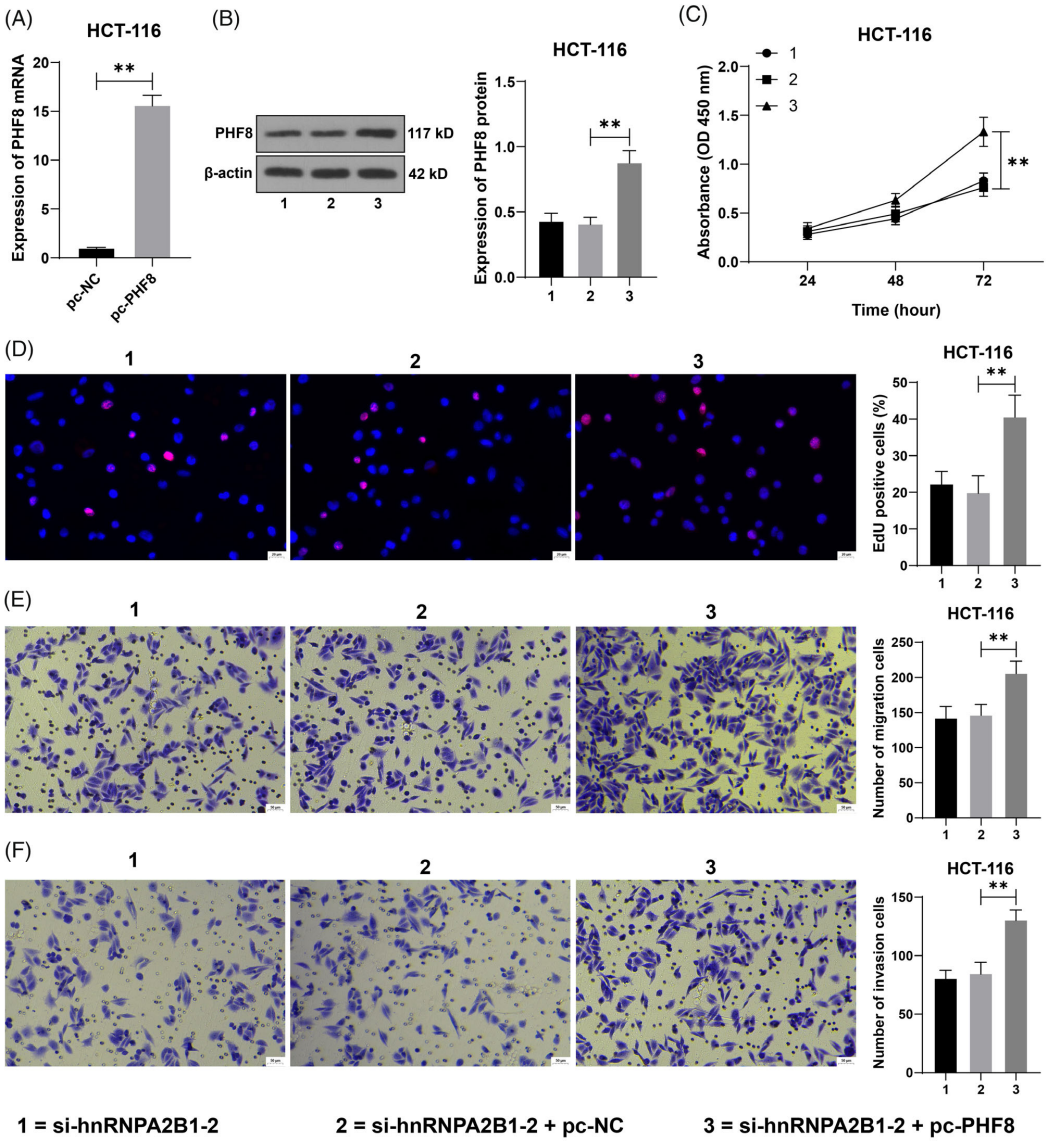
Overexpression of PHF8 reduces the inhibitory effect of hnRNPA2B1 silencing on CRC cells. PHF8 pcDNA3.1 (pc‐PHF8) was transfected into HCT‐116 cells, with empty pcDNA3.1 (pc‐NC) as a negative control. (A,B) PHF8 expression in cells was detected by RT‐qPCR and Western blot. (C) The cell proliferation was evaluated by CCK‐8 assay. (D) The percentage of EdU‐positive cells was detected by EdU staining. (E,F) Transwell detected cell invasion and migration. The cell experiments were repeated three times independently. Data are presented as mean ± standard deviation. Data in penal (A) (right) were analyzed using a *t*‐test. Data in panels (B,D–F) were analyzed by one‐way ANOVA and data in panel (C) was analyzed by two‐way ANOVA, followed by Tukey's multiple comparisons tests. **p < 0.01.

The current investigation effectively enhanced PHF8 expression in HCT‐116 cells using the transfection of pc‐PHF8, subsequently integrating the experiment with si‐hnRNPA2B1‐2 (*p* < 0.05, Figure 6A,B). The results demonstrated that overexpression of PHF8 led to enhanced cell proliferation activity, increased percentage of EdU‐positive cells (*p* < 0.05, Figure 6C,D), and augmented migration and invasion (*p* < 0.05, Figure 6E,F). The above results indicated that overexpression of PHF8 alleviated the inhibitory effect of hnRNPA2B1 silencing on CRC cells.

## 4 DISCUSSION

**FIGURE 7:**
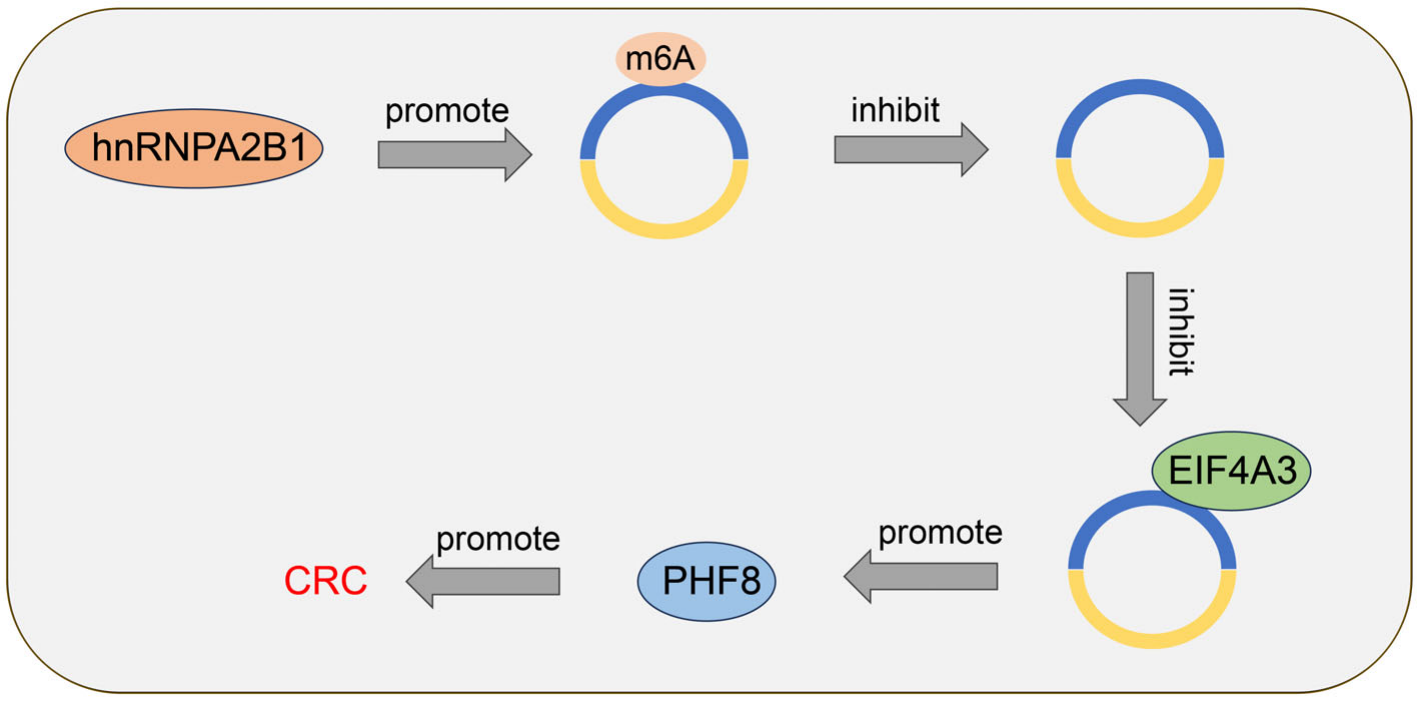
hnRNPA2B1 promotes CRC cell progression via the circCDYL/PHF8 axis. hnRNPA2B1 reduces the expression of circCDYL by enhancing circCDYL m6A modification level, thereby reducing its binding to EIF4A3 and promoting the expression of PHF8, ultimately accelerating the progression of colorectal cancer.

A significant percentage of CRC patients may still experience recurrence and metastasis despite the increased survival, resulting in improvements in disease knowledge and treatment.^30^ Overexpression of hnRNPA2B1 is found in many cancers and is closely associated with several malignant characteristics, including angiogenesis, metastasis, and treatment resistance.^31^ This study elucidates that hnRNPA2B1 drives CRC progression via the circCDYL/EIF4A3/PHF8 axis (Figure 7).

Emerging studies have unveiled that hnRNPA2B1 overexpression is associated with oncogenesis in CRC.^21^, ^23^, ^32^ hnRNPA2B1 directs CRC progression by inducing EMT and metastasis.^33^ The CRNDE/hnRNPA2B1 axis specifically activates MAPK signaling by enhancing the translation of KRAS mRNA, thereby accelerating the malignant progression of CRC.^18^ The results consistently demonstrated a high expression pattern of hnRNPA2B1 in CRC cells, and the silencing of hnRNPA2B1 inhibited CRC cell proliferation, invasion, and migration.

Furthermore, hnRNPA2B1 functions as a m6A reader, influencing a complex range of RNA metabolic processes.^17^ m6A serves as a reversible epigenetic modification in mRNAs and ncRNAs, affecting the fate of modified RNAs and regulating key biological processes, including carcinogenesis and cancer progression.^15^ Growing evidence has unveiled the presence of m6A modification in circRNAs and the cross‐talk between circRNAs and m6A modification in diverse pathophysiological processes.^34^ Previous studies have indicated a suppressive role of circCDYL in CRC growth and metastasis,^14^ and circCDYL undergoes m6A RNA methylation.^24^ This study, therefore, hypothesized that circCDYL, as a downstream mechanism of hnRNPA2B1, contributed to the advancement of CRC. These findings showed that hnRNPA2B1 knockdown increased circCDYL expression while circCDYL expression decreased in CRC cells. The present study then investigated whether m6A mRNA modification was necessary for hnRNPA2B1 to regulate circCDYL expression. The m6A level of circCDYL in cells significantly decreased following si‐hnRNPA2B1 treatment. The findings demonstrate that hnRNPA2B1 reduces circCDYL expression by promoting m6A modification of circCDYL in CRC cells. circCDYL reduces the viability and increases the apoptosis of colon cancer cells by inhibiting miR‐150‐5p.^14^ Low expression of circCDYL has been shown to mitigate the inhibitory effect of hnRNPA2B1 silencing on CRC cells.

The present study eventually focused on investigating the downstream target regulated by circCDYL in CRC. It has been hypothesized that circCDYL may interact with the RNA‐binding protein EIF4A3, as indicated by the Circintractome database. EIF4A3, a fundamental element of the exon junction complex, functions as an RNA binding protein that coordinates mRNA splicing and affects subsequent processes.^35^ EIF4A3 can manipulate the expression of circRNAs by controlling the back splicing of circRNAs in CRC. For example, circCOL1A1 expedites angiogenesis of human umbilical vein endothelial cells by recruiting EIF4A3 protein in CRC.^36^ EIF4A3 induces circ_0084615 expression, accelerating CRC cell growth, motility, and angiogenesis.^37^ The binding of circ_cse1l to EIF4A3 downregulates the expression of PCNA, thus impeding the proliferation of CRC cells.^38^ The current research used RIP and RNA pull‐down experiments to validate the interaction between circCDYL and EIF4A3.

Furthermore, EIF4A3 was predicted to interact with PHF8 in the Starbase database. PHF8, a histone lysine demethylase belonging to the Jumonji C protein family, is significantly involved in various cellular and molecular processes.^39^ PHF8 expression is increased in CRC, and higher PHF8 expression is predictive of poorer overall survival.^29^ Ablation of PHF8 impedes CRC growth, augments antiviral immune responses, and sensitizes immune checkpoint blockade therapy in a mouse model of CRC.^40^ Elevated PHF8 expression has also been observed in CRC cells. RIP analysis demonstrated that PHF8 mRNA was co‐precipitated by EIF4A3, and the enrichment of PHF8 mRNA in EIF4A3 precipitation was increased following the knockdown of circCDYL. Moreover, the binding of circCDYL to EIF4A3 diminished the expression of PHF8. Knockdown of PHF8 dramatically depresses CRC cell proliferation and migration but expedites apoptosis.^29^ The functional rescue tests demonstrated that the overexpression of PHF8 reversed the inhibitory impact of hnRNPA2B1 silencing on CRC cells.

In summary, hnRNPA2B1‐mediated m6A alteration reduces circCDYL expression, inhibiting circCDYL's interaction with EIF4A3, enhancing PHF8 expression, and accelerating CRC progression. This study also has certain limitations. This study is limited to the cellular level, clinical samples could not be obtained for examination due to funding constraints. The current study primarily investigated the mechanisms downstream of hnRNPA2B1 without examining those upstream of hnRNPA2B1. Furthermore, this study has only validated the function of a single mechanism in two CRC cell lines, and it remains uncertain whether our mechanism applies to other cell types. In addition to hnRNPA2B1, the potential synergistic contributions of other m6A functional enzymes to CRC advancement remain unclear. In the future, clinical samples will be acquired to ascertain the clinical significance of hnRNPA2B1 in CRC. Furthermore, the upstream mechanisms of hnRNPA2B1 will be investigated and the role of other m6A functional enzymes will be analyzed to contribute new theoretical insights for CRC treatment.

## CONFLICT OF INTEREST STATEMENT

All authors declare no conflicts of interest.

## Data Availability

The data that support the findings of this study are available from the corresponding author upon reasonable request.

## References

[1] Kim BJ , Hanna MH . Colorectal cancer in young adults. J Surg Oncol. 2023;127(8):1247–1251. 37222697 10.1002/jso.27320

[2] Ahmad R , Singh JK , Wunnava A , Al‐Obeed O , Abdulla M , Srivastava SK . Emerging trends in colorectal cancer: dysregulated signaling pathways (review). Int J Mol Med. 2021;47(3):14. 33655327 10.3892/ijmm.2021.4847 PMC7834960

[3] Xu W , He Y , Wang Y , Li X , Young J , Ioannidis JPA , et al. Risk factors and risk prediction models for colorectal cancer metastasis and recurrence: an umbrella review of systematic reviews and meta‐analyses of observational studies. BMC Med. 2020;18(1):172. 32586325 10.1186/s12916-020-01618-6 PMC7318747

[4] Dekker E , Tanis PJ , Vleugels JLA , Kasi PM , Wallace MB . Colorectal cancer. Lancet. 2019;394(10207):1467–1480. 31631858 10.1016/S0140-6736(19)32319-0

[5] Geuens T , Bouhy D , Timmerman V . The hnRNP family: insights into their role in health and disease. Hum Genet. 2016;135(8):851–867. 27215579 10.1007/s00439-016-1683-5 PMC4947485

[6] Jiang F , Tang X , Tang C , Hua Z , Ke M , Wang C , et al. HNRNPA2B1 promotes multiple myeloma progression by increasing AKT3 expression via m6A‐dependent stabilization of ILF3 mRNA. J Hematol Oncol. 2021;14(1):54. 33794982 10.1186/s13045-021-01066-6 PMC8017865

[7] Ayoufu A , Yi L , Tuersuntuoheti M , Li Y . HNRNPA2B1 is a potential biomarker of breast cancer related to prognosis and immune infiltration. Aging (Albany NY). 2023;15(17):8712–8728. 37671941 10.18632/aging.204992 PMC10522385

[8] Li K , Gong Q , Xiang XD , Guo G , Liu J , Zhao L , et al. HNRNPA2B1‐mediated m(6)A modification of lncRNA MEG3 facilitates tumorigenesis and metastasis of non‐small cell lung cancer by regulating miR‐21‐5p/PTEN axis. J Transl Med. 2023;21(1):382. 37308993 10.1186/s12967-023-04190-8 PMC10258935

[9] Tang J , Chen Z , Wang Q , Hao W , Gao WQ , Xu H . hnRNPA2B1 promotes colon cancer progression via the MAPK pathway. Front Genet. 2021;12:666451. 34630502 10.3389/fgene.2021.666451 PMC8494201

[10] Shao R , Zhang Z , Xu Z , Ouyang H , Wang L , Ouyang H , et al. H3K36 methyltransferase NSD1 regulates chondrocyte differentiation for skeletal development and fracture repair. Bone Res. 2021;9(1):30. 34099628 10.1038/s41413-021-00148-y PMC8185073

[11] Chen L , Shan G . CircRNA in cancer: fundamental mechanism and clinical potential. Cancer Lett. 2021;505:49–57. 33609610 10.1016/j.canlet.2021.02.004

[12] Liang G , Ling Y , Mehrpour M , Saw PE , Liu Z , Tan W , et al. Autophagy‐associated circRNA circCDYL augments autophagy and promotes breast cancer progression. Mol Cancer. 2020;19(1):65. 32213200 10.1186/s12943-020-01152-2 PMC7093993

[13] Sun J , Zhang H , Tao D , Xie F , Liu F , Gu C , et al. CircCDYL inhibits the expression of C‐MYC to suppress cell growth and migration in bladder cancer. Artif Cells Nanomed Biotechnol. 2019;47(1):1349–1356. 30968727 10.1080/21691401.2019.1596941

[14] Cui W , Dai J , Ma J , Gu H . circCDYL/microRNA‐105‐5p participates in modulating growth and migration of colon cancer cells. Gen Physiol Biophys. 2019;38(6):485–495. 31829306 10.4149/gpb2019037

[15] Huang H , Weng H , Chen J . m(6)A modification in coding and non‐coding RNAs: roles and therapeutic implications in cancer. Cancer Cell. 2020;37(3):270–288. 32183948 10.1016/j.ccell.2020.02.004 PMC7141420

[16] Qiao H , Liu L , Chen J , Shang B , Wang L . The functions of N6‐methyladenosine (m6A) RNA modifications in colorectal cancer. Med Oncol. 2022;39(12):235. 36175777 10.1007/s12032-022-01827-4

[17] Alarcon CR , Goodarzi H , Lee H , Liu X , Tavazoie S , Tavazoie SF . HNRNPA2B1 is a mediator of m(6)A‐dependent nuclear RNA processing events. Cell. 2015;162(6):1299–1308. 26321680 10.1016/j.cell.2015.08.011 PMC4673968

[18] Lu Y , Zou R , Gu Q , Wang X , Zhang J , Ma R , et al. CRNDE mediated hnRNPA2B1 stability facilitates nuclear export and translation of KRAS in colorectal cancer. Cell Death Dis. 2023;14(9):611. 37716979 10.1038/s41419-023-06137-9 PMC10505224

[19] Li Y , Li K , Lou X , Wu Y , Seery S , Xu D , et al. HNRNPA2B1‐mediated microRNA‐92a upregulation and section acts as a promising noninvasive diagnostic biomarker in colorectal cancer. Cancers (Basel). 2023;15(4):1367. 36831695 10.3390/cancers15051367 PMC9954252

[20] Zhao S , Mi Y , Guan B , Zheng B , Wei P , Gu Y , et al. Correction to: tumor‐derived exosomal miR‐934 induces macrophage M2 polarization to promote liver metastasis of colorectal cancer. J Hematol Oncol. 2021;14(1):33. 33618743 10.1186/s13045-021-01042-0 PMC7901099

[21] Liu H , Li D , Sun L , Qin H , Fan A , Meng L , et al. Interaction of lncRNA MIR100HG with hnRNPA2B1 facilitates m(6)A‐dependent stabilization of TCF7L2 mRNA and colorectal cancer progression. Mol Cancer. 2022;21(1):74. 35279145 10.1186/s12943-022-01555-3 PMC8917698

[22] Livak KJ , Schmittgen TD . Analysis of relative gene expression data using real‐time quantitative PCR and the 2(‐Delta Delta C(T)) method. Methods. 2001;25(4):402–408. 11846609 10.1006/meth.2001.1262

[23] Li Y , Wang H , Wan J , Ma Q , Qi Y , Gu Z . The hnRNPK/A1/R/U complex regulates gene transcription and translation and is a favorable prognostic biomarker for human colorectal adenocarcinoma. Front Oncol. 2022;12:845931. 35875075 10.3389/fonc.2022.845931 PMC9301189

[24] Wei Y , Fu J , Zhang H , Ling Y , Tang X , Liu S , et al. N6‐methyladenosine modification promotes hepatocarcinogenesis through circ‐CDYL‐enriched and EpCAM‐positive liver tumor‐initiating exosomes. iScience. 2023;26(10):108022. 37954137 10.1016/j.isci.2023.108022 PMC10638478

[25] Liu H , Lan T , Li H , Xu L , Chen X , Liao H , et al. Circular RNA circDLC1 inhibits MMP1‐mediated liver cancer progression via interaction with HuR. Theranostics. 2021;11(3):1396–1411. 33391541 10.7150/thno.53227 PMC7738888

[26] Dudekula DB , Panda AC , Grammatikakis I , De S , Abdelmohsen K , Gorospe M . CircInteractome: a web tool for exploring circular RNAs and their interacting proteins and microRNAs. RNA Biol. 2016;13(1):34–42. 26669964 10.1080/15476286.2015.1128065 PMC4829301

[27] Li JH , Liu S , Zhou H , Qu LH , Yang JH . starBase v2.0: decoding miRNA‐ceRNA, miRNA‐ncRNA and protein‐RNA interaction networks from large‐scale CLIP‐Seq data. Nucleic Acids Res. 2014;42(Database issue):D92–D97. 24297251 10.1093/nar/gkt1248 PMC3964941

[28] Wu XN , Li JY , He Q , Li BQ , He YH , Pan X , et al. Targeting the PHF8/YY1 axis suppresses cancer cell growth through modulation of ROS. Proc Natl Acad Sci U S A. 2024;121(2):e2219352120. 38165927 10.1073/pnas.2219352120 PMC10786316

[29] Lv Y , Shi Y , Han Q , Dai G . Histone demethylase PHF8 accelerates the progression of colorectal cancer and can be regulated by miR‐488 in vitro. Mol Med Rep. 2017;16(4):4437–4444. 28765946 10.3892/mmr.2017.7130 PMC5647003

[30] Haria PD , Baheti AD , Palsetia D , Ankathi SK , Choudhari A , Guha A , et al. Follow‐up of colorectal cancer and patterns of recurrence. Clin Radiol. 2021;76(12):908–915. 34474747 10.1016/j.crad.2021.07.016

[31] Chen C , Huang L , Sun Q , Yu Z , Wang X , Bu L . HNRNPA2B1 demonstrates diagnostic and prognostic values based on pan‐cancer analyses. Comput Math Methods Med. 2022;2022:9867660. 35529270 10.1155/2022/9867660 PMC9068287

[32] Zhang Y , Huang W , Yuan Y , Li J , Wu J , Yu J , et al. Correction to: Long non‐coding RNA H19 promotes colorectal cancer metastasis via binding to hnRNPA2B1. J Exp Clin Cancer Res. 2021;40(1):111. 33757526 10.1186/s13046-021-01911-z PMC7988987

[33] Hu HF , Han L , Fu JY , He X , Tan JF , Chen QP , et al. LINC00982‐encoded protein PRDM16‐DT regulates CHEK2 splicing to suppress colorectal cancer metastasis and chemoresistance. Theranostics. 2024;14(8):3317–3338. 38855188 10.7150/thno.95485 PMC11155395

[34] Liu F , Gu W , Shao Y . Cross‐talk between circRNAs and m6A modifications in solid tumors. J Transl Med. 2024;22(1):694. 39075555 10.1186/s12967-024-05500-4 PMC11288061

[35] Kanellis DC , Espinoza JA , Zisi A , Sakkas E , Bartkova J , Katsori AM , et al. The exon‐junction complex helicase eIF4A3 controls cell fate via coordinated regulation of ribosome biogenesis and translational output. Sci Adv. 2021;7(32):eabf7561. 34348895 10.1126/sciadv.abf7561 PMC8336962

[36] Hu G , Lin C , Gao K , Chen M , Long F , Tian B . Exosomal circCOL1A1 promotes angiogenesis via recruiting EIF4A3 protein and activating Smad2/3 pathway in colorectal cancer. Mol Med. 2023;29(1):155. 37940881 10.1186/s10020-023-00747-x PMC10633966

[37] Jiang Z , Tai Q , Xie X , Hou Z , Liu W , Yu Z , et al. EIF4A3‐induced circ_0084615 contributes to the progression of colorectal cancer via miR‐599/ONECUT2 pathway. J Exp Clin Cancer Res. 2021;40(1):227. 34253241 10.1186/s13046-021-02029-y PMC8273970

[38] Xu B , Yang N , Liu Y , Kong P , Han M , Li B . Circ_cse1l inhibits colorectal cancer proliferation by binding to eIF4A3. Med Sci Monit. 2020;26:e923876. 32857753 10.12659/MSM.923876 PMC7477927

[39] Qi HH , Sarkissian M , Hu GQ , Wang Z , Bhattacharjee A , Gordon DB , et al. Histone H4K20/H3K9 demethylase PHF8 regulates zebrafish brain and craniofacial development. Nature. 2010;466(7305):503–507. 20622853 10.1038/nature09261 PMC3072215

[40] Liu Y , Hu L , Wu Z , Yuan K , Hong G , Lian Z , et al. Loss of PHF8 induces a viral mimicry response by activating endogenous retrotransposons. Nat Commun. 2023;14(1):4225. 37454216 10.1038/s41467-023-39943-y PMC10349869

